# Isolation, Identification, and Genetic Characterization of Antibiotic Resistance of *Salmonella* Species Isolated from Chicken Farms

**DOI:** 10.1155/2022/6065831

**Published:** 2022-11-29

**Authors:** Ahmed Shalaby, Mahmoud M. Ismail, Hanem El-Sharkawy

**Affiliations:** Department of Poultry and Rabbit Diseases, Faculty of Veterinary Medicine, Kafrelsheikh University, Kafrelsheikh 33511, Egypt

## Abstract

*Salmonella* is a major cause of foodborne outbreaks. It causes gastroenteritis in humans and animals. This micro-organism causes severe illness in chickens and has a major impact on chicken productivity and the poultry industry. This study aimed to address the prevalence of *Salmonella* infection in broiler chicken farms in Kafrelsheikh, Gharbia, and Menofeya provinces in Egypt during 2020–2022. This work also aimed to evaluate the genetic characterization and antibiotic resistance of the isolated *Salmonella* strains. Clinical signs and mortalities were observed and recorded. In total, 832 samples were collected from 52 broiler flocks, including 26 from both one-week-old and 6-week-old chicken farms from different organs (liver, intestinal content, spleen, and gallbladder). The prevalence of *Salmonella* infections was reported in the study region to be 36.54%. Of the 26 one-week-old farms surveyed, 11 (42.31%) and 8/26 (30.77%) of the six-week-old broiler chicken farms had *Salmonella* infections. Recovered isolates were serotyped as 9 (47.37%) *S. enteritidis* O 1,9,12, ad monophasic H: g, m: -, 6 (31.58.%) *S. shangani* 2, (10.53%) *S. gueuletapee* 1, (5.26%) *S. II (salamae)*, and 1 (5.26%) untypable. The results showed that *Salmonella* infection was predominant in one-week-old chicks compared to infection in six-week-old and uninfected flocks. All *Salmonella* isolates were resistant to ampicillin and erythromycin, while all isolates were sensitive to ciprofloxacin, chloramphenicol, and levofloxacin. The isolates also contained 10.53% (2/19) streptomycin, 10.53% (2/21) gentamicin, 15.79% (3/19) doxycycline, and 26.32% (5/19) lincomycin and colistin. The phenotypically resistant *Salmonella* samples against ampicillin, erythromycin, and macrolide harbored *bla*_TEM_*, bla*_SHV_*, erm*B*, ere*A*, mph*A, and *erm*B, respectively. This baseline data on *Salmonella* spp. prevalence, serotyping, and antibiotic profiles are combined to define the antimicrobial resistance to this endemic disease. Elucidation of the mechanisms underlying this drug resistance should be of general importance in understanding both the treatment and prevention of *Salmonella* infection in this part of Egypt.

## 1. Introduction

Chicken is a significant source of eggs and meat. The poultry production-related industry is one of the economically important agro-industry components [1].*Salmonella* spp. is one of the most causative agents of diseases in poultry and avian species [2]. It causes heavy economic losses because of its high mortality rate and reduced production rate in poultry [3]. The economic cost of the *Salmonella* spp. outbreak has been estimated to be $11.6 billion in the USA [4] and more than €3 billion in the European Union [5].

Poultry is one of the most preferable reservoirs for *Salmonella* spp., which will allow it to transmit to humans through food [6]. Poultry meat is thought to be the most common source of *Salmonella* infection in humans, accounting for roughly 40% of clinically reported cases [7].

*Salmonella* spp. is a Gram-negative bacteria that belongs to the family Enterobacteriaceae [8]. *Salmonella* spp. is an opportunistic zoonotic organism that infects human and animal cells through contaminated food and the environment [9]. It infects a wide variety of cells, such as M cells, epithelial cells, dendritic cells, and macrophages [10]. It can survive both in the absence and presence of oxygen [11].

*Salmonella* spp. chickens are usually divided into three classes based on the diseases they cause [12]. The first class comprises nonmotile, chicken-adapted*Salmonella*e, which include *S. gallinarum*, which causes fowl typhoid, and *S. pullorum*, which causes pullorum disease in chickens [13]. Fowl typhoid (FT) and pullorum disease (PD) are septicemic diseases that are usually most significant in growing and adult chickens. In mature poultry, symptoms of FT and PD include reduced egg production, reduced fertility, decreased hatchability, anorexia, and increased mortality [14].

The second class of *Salmonella* that affects birds is the invasive, nonhost-specific*Salmonella*, which can infect more than one host, including animals and humans, and is called paratyphoid *Salmonella* in birds. This type of bacteria causes paratyphoid in birds, and it is of zoonotic concern. The paratyphoid *Salmonella* includes 10–20 serovars. *S. enteritidis, S. typhimurium, S. shangani, S. gueuletapee*, and *S. II salamae* are the most important serovars [15].

*Salmonella enteritidis, S. typhimurium,* S. *shangani*, *S. gueuletapee*, and S. *II salamae* have been reported to be the most common *salmonellae* isolated from Egyptian poultry farms [16]. They are transmitted horizontally between farms and vertically to the progeny through trans-ovarian infection [16]. The clinical manifestations of paratyphoid infections are most predominant in young chickens, especially in the first few weeks of life. The most common symptoms associated with paratyphoid disease in broilers include depression, anorexia, and diarrhea, with high mortalities, especially in the first week of life. While in adult birds, the infection is asymptomatic, and the infected birds are considered carriers, which are the most dangerous source for the shedding of bacteria in meat and eggs of zoonotic concern (Tiwari, Swamy, et al.).

The third class of *Salmonella* is neither host-adapted nor invasive and may cause disease in humans and other animals [14].

The widespread use of antibiotics on poultry farms as growth promoters or prophylactics as well as for treatment can raise concerns about antibiotic resistance, which has been reported in many *Salmonella* spp. serovars [17]. During the second half of the twentieth century, there were two significant breakthroughs in the epidemiology of nontyphoidal salmonellosis throughout the world [18]. First, multidrug-resistant*Salmonella typhimurium* strains, such as *S. typhimurium* DT104, have arisen, and second, *Salmonella* enteritidis has emerged as a prominent poultry and egg pathogen [19].

Amoxicillin (*β*-lactam antibiotic) competitively inhibits penicillin-binding protein 1. By producing an enzyme called a *β*-lactamase, which attacks the *β* -lactam ring; bacteria frequently become resistant to *β*-lactam antibiotics. Prophylactic *β*-lactam resistance develops through four main mechanisms: the production of a *β*-lactamase enzyme (primarily in Gram-negative bacteria), low expression of external membrane proteins, alterations in the dynamic site of penicillin-binding proteins (PBPs), and active efflux [20]. There are genes that are associated with resistance to *β*-lactamase*bla*_TEM-1_, *bla*_TEM-2_, and *bla*_SHV-1_. The *β*-lactam ring of penicillin is hydrolyzed by *bla*_TEM_*β*-lactamases, which is how they work. There are three types of SHVs (sulfhydryl variables): 2b, 2be, and 2ber. Penicillin and first- and second-generation cephalosporins are hydrolyzed by type 2b; third-generation cephalosporins are hydrolyzed by type 2be; while clavulanic acid and tazobactam are resistant to type 2br. Every year, new *β*-lactamase variants are recorded, and this poses a challenge to the medical field [21].

Erythromycin stops bacteria from producing their protein by attaching to the bacterial cell membrane and the 50S subunit of the ribosome. A small 30S subunit and a large 50S subunit make up the bacteria's ribosome. The latter has at least 30 proteins and 23S rRNA. Erythromycin inhibits protein synthesis by attaching to the 50S subunit. Erythromycin ribosomal methylase is a ribosomal enzyme that modifies the 50S subunit's binding site for erythromycin. It is encoded by the ermB gene. The modification gene markedly reduces the affinity of erythromycin for its target [22]. Macrolides, including erythromycin, inhibit bacterial protein synthesis by binding at the exit tunnel of the 50S ribosomal subunit. They do this by preventing peptidyl transferase from adding the growing peptide attached to tRNA to the next amino acid. It also inhibits bacterial ribosomal translation [23]. Macrolide inactivation also occurs by phosphotransferases encoded by *mphA* and *mphB* [24]. A resistance enzyme that preferentially inactivates 14-membered macrolides (such as erythromycin, telithromycin, and roxithromycin) over 16-membered macrolides is encoded by the mphA gene (e.g., tylosin and spiramycin). It phosphorylates macrolides in a GTP-dependent manner at the 2′-OH hydroxyl group of the desosamine sugar of macrolides [25]. Resistance to macrolides may also be due to the *ereA* gene (erythromycin resistance esterase type I) [26]. This encodes the erythromycin esterase enzyme, which causes enzymatic hydrolysis of the macrolactone ring [23].

Serotyping is a basic biomarker for investigating the epidemiology status of *Salmonella* infections, and it's frequently used to allocate the source of contamination during epidemics [27]. This method was established by White and Kauffmann based on the detected phase-shift flagella antigen and flagella H, somatic O antigen [2]. The method addressed is considered the reference one for the serotyping of *Salmonella* spp. Serotyping of *Salmonella* spp. has many advantages, including details regarding the disease's severity, the source of contamination, and the pattern of resistance. Molecular characterization methods have been used to identify differences between *Salmonella* strains. These methods include PCR, pulsed-field gel electrophoresis (PFGE), random amplification of polymorphic DNA (RAPD), etc. [28].

This study aimed to isolate and identify *Salmonella* spp. from different provinces in Egypt. The study also concludes the investigation of antimicrobial resistance against 11 different clinically relevant antimicrobials and the molecular characterization of resistance-attributed genes.

## 2. Methods

### 2.1. Sampling Strategy and *Salmonella* Isolation

This study has conveniently targeted 52 broiler chicken flocks (Avian 48, Abdelsalam Hegazy Company), of which 26 were one-week-old chick flocks and 26 were six-week-old birds. These farms were investigated for *Salmonella* infections. The broiler chicken flocks were surveyed in Kafrelsheikh, Gharbia, and Menofeya provinces in Egypt during 2020–2022. The birds showed different clinical signs, including reluctance to move, pasty diarrhea, huddling near the source of the heat, ruffling feathers, dehydration, decreased body weight gain, droopy wings, lameness, and high mortalities of 9.64% ± 1.72 in one-week-old chicks from each broiler chicken farm. Four living, diseased birds were selected randomly and sacrificed. At postmortem, sections from the liver, gallbladder, spleen, and intestinal contents were collected under aseptic conditions for *Salmonella* isolation. Within five hours of collection, samples were delivered to the lab and stored on ice until then. Selenite-F broth (SFB) (Oxoid, UK) was combined with one gramme of tissue from each organ and incubated statically at 37°C for an overnight period. The enrichments were applied to XLD agar (Oxoid, UK) using a swap, and they were then incubated at 37°C overnight. One colony from each plate that appeared to be *Salmonella* spp. was chosen for additional examinations based on appearance [29].

### 2.2. Biochemical Identification

The pure pink colonies on XLD agar with black center colouration were taken as suspected colonies of *Salmonella* spp. According to Lamboro et al. [30], these bacterial colonies were confirmed biochemically as *Salmonella* spp. [30]. The biochemical tests used for *Salmonella* spp. detection were IMViC reactions that included indole, methyl red, Vogues Proskauer, oxidase, and citrate utilization tests [31]. Urease hydrolysis and hydrogen peroxide production were also tested [31].

### 2.3. Serological Identification of *Salmonella* Isolates

Serotyping of suspected *Salmonella* strains was conducted at the Animal Health Research Institute, Dokki, Giza, Egypt, according to the manufacturer's instructions (Denka Seiken Co., Tokyo, Japan). Briefly, the isolates were examined with an omnivalent A-67. The positive isolates were tested with anti-*Salmonella* A-E and anti-*Salmonella* F-67. The samples were identified by using anti-*Salmonella* antibodies grouped by specific O antigens (2, 4, 7, 8, etc.). The samples were tested for grouped anti-*Salmonella* H antigen phases 1 and 2.

### 2.4. Genomic DNA Extraction and Purification

A single colony was collected from each plate and inoculated into five ml of selenite-F broth SFB (Oxoid, UK) throughout the course of an overnight period at 37°C. One minute of 13000 rpm centrifugation was performed on one milliliter of bacterial culture broth in a microcentrifuge tube. After removing the supernatant, the bacterial pellets were heated at 95°C for 10 minutes while being homogenized with water devoid of nucleases. Finally, the boiled lysates were centrifuged, and the supernatant was removed to create DNA templates that were stored at −80°C until use [32].

### 2.5. Molecular Detection of the *Salmonella* Genus and Antimicrobial Resistance-Associated Genes

The *ompC* gene was used as a specific determinant for *Salmonella* spp. detection [33]. The amplification of *ompC* PCR was performed using primers, as shown in Table 1, according to the method described by the authors of [33]. *Salmonella* isolates were screened for five genes known to be associated with antibiotic resistance to ampicillin, erythromycin, and macrolides. These genes are *bla*_TEM_, *bla*_SHV_, and *erm*B, *ere*A, and *mph*A, respectively, as shown in Table 1 according to the methods described by [34, 35].

Briefly, primers were utilized in a 25 *μ*l of uniplex PCR mix, comprising 12.5 *μ*l of EmeraldAmp Max PCR Master Mix (Takara, Japan), 1 *μ*l of each primer (20 pmol), 5.5 *μ*l of water, and 5 *μ*l of DNA template. The reaction was performed in an Applied Biosystems 2720 thermal cycler. The cycling condition started with primary denaturation at 94°C for 5 min, followed by 35 cycles and a final extension at 72°C for 10 min. The specific annealing of each gene is shown in Table 1. The positive controls were represented by field samples that were previously confirmed to be positive by PCR for the antimicrobial resistance-related genes in the reference laboratory for veterinary quality control on poultry production, an Animal Health Research Institute. The *Salmonella* ATCC 9184 strain was used as a control positive for *ompC* gene detection, while sterile water was added to the PCR mix with each primer pair as a control negative.

### 2.6. The Antimicrobial Susceptibility Test

Antimicrobial susceptibility testing (AST) was carried out using the Kirby–Bauer disc diffusion method as recommended by the CLSI [36]. *E. coli* ACTC25922 and *E. coli* NCTC10418 were used as quality control strains during AST. The AST for *Salmonella* isolates was conducted against 11 antimicrobial agents that are clinically used in the Egyptian poultry industry. This includes ciprofloxacin (CIP 5 *μ*g), chloramphenicol (C 30 *μ*g), streptomycin (STR 10 *μ*g), gentamicin (CN 10 *μ*g), erythromycin (E 15 *μ*g), doxycycline (DO 30 *μ*g), levofloxacin (LEV 5 *μ*g), ampicillin (AM 10 *μ*g), lincomycin (L 2 *μ*g), norfloxacin (NOR 10 *μ*g), and colistin (CT 10 *μ*g). The tested *Salmonella* inoculum was prepared by direct saline suspension of a nutrient broth culture from an isolated colony on selective XLD agar plates that had been incubated for 18 to 24 hours. The bacterial suspension of tested *Salmonella* was adjusted in sterile saline by adding approximately one ml of overnight bacterial suspension to 4 ml of sterile saline to match the 0.5 McFarland standard (containing approximately 1–2 x 10^8^ CFU/ml for American Type Culture Collection (ATCC) 2592 *E. coli*) by using a McFarland densitometer (Biomerieux Biotechnology, UK). Using a sterile swab, the saline suspension was applied to the Mueller–Hinton Agar plate (Oxoid, UK). Antibiotic-containing antimicrobial discs were strewn throughout the Mueller–Hinton agar surface after it had been inoculated. Overnight, the agar plates were incubated at 37°C. Using sliding calipers and interpretation, the diameters of the inhibited zones, including the diameter of the discs, were measured and observed according to the Clinical Laboratory Standards Institute (Table 2) [37].

### 2.7. Statistical Analysis

Student's *t*-tests were employed using Microsoft Excel software for the percentage of mortalities related to *Salmonella* infection and the rate of isolation of *Salmonellae* from internal organs, according to the method described by [38].

## 3. Results

### 3.1. Clinical Signs, Incidence, and Mortalities of *Salmonella* spp

Samples were collected from 52 broiler chicken farms from the study regions, and clinical symptoms of *Salmonella* infection were gathered at the time of sampling. The symptoms, which included diarrhea, dehydration, decreased body weight gain, lameness, and significant mortalities, were primarily seen in one-week-old broiler chicks. Hepatitis, hepatomegaly with necrotic foci, arthritis, typhlitis, omphalitis, myocarditis, and pneumonia were the predominant postmortem pathologies. However, the symptoms were less severe in older birds at the 6^th^ week of age.

Out of 832 clinical samples collected from 52 broiler flocks from different organs (liver, intestinal content, spleen, and gallbladder), 19 (2.28%) putative *Salmonella* spp. were isolated from individual birds (one isolate per bird) (Table 3).

Of all the 26 surveyed one-week-old farms, 11 (42.31%) and 8/26 (30.77%) of the six-week-old broiler chicken farms had *Salmonella* infection. In the first-week-old birds, *Salmonella* infection caused a significantly higher (*P* < 0.01) mortality rate in the *Salmonella*-positive flocks (9.64% ± 1.72) compared to the negative flocks (2.5% ± 0.99). However, the mortality rates of the infected and uninfected 6-week-old flocks did not significantly differ (*P*=0.15). The rates of isolation of *Salmonella* from the liver 7/208 (3.36%) and gallbladder 6/208 (2.88%) were significantly (*P* < 0.05) higher than those isolated from the spleen and intestinal content, with an isolation rate of 3/208 (1.44%) for both of them (Table 3).

### 3.2. Molecular Identification of *Salmonella* Isolates

**Figure 1:**
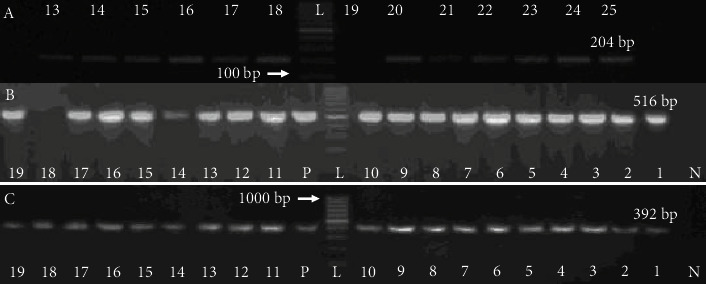
*Salmonella spp.* and beta-lactam resistance genes are diagnostic. PCR amplification of the (a) *ompC* (representative), the (b–c) *β*-lactam resistance genes *bla*_TEM_ and *bla*_SHV_ are found in *Salmonella spp.* Electrophoresis was carried out on a 1.5% agarose gel. Lane L 100 bp DNA ladder, lane P for positive control, and lane N for negative control. Samples were run on a 1.5% TAE agarose gel.

The isolates were confidently identified as *Salmonella* spp. by amplification of the *ompC* gene (Figure 1). The PCR confirmed 19 of the *Salmonella* isolates that were identified phenotypically and biochemically.

### 3.3. Results of Serological Identification of *Salmonella* Isolates

The isolated *Salmonellae* (*n* = 19) were serotyped. Our finding showed that *S. enteritidis* 9 (47.37%) with *O* antigen 1,9,12 and H antigen phase one g, m. *S. shangani* 6 (31.58.%) with O antigen 3,10,15 and H antigen, phase one *d*, and phase two 1, 5. *S. gueuletapee* 2 (10.53%) with O antigen 9,12, and H antigen phase 1 g,m,s; *S. II (salamae)* 1 (5.26%) with O antigen 6,8 and H antigen phase one g,s,t; and H antigen phase two e,n,x; and untypable *Salmonella* 1 (5.26%) (Table 4**)**.

### 3.4. Antimicrobial Resistance Profiles

*Salmonella* spp. resistance to *β*-lactamase ampicillin was 100% (19/19), ciprofloxacin 0% (0/19), erythromycin 100% (19/19), chloramphenicol 0% (0/19), streptomycin 10.53% (2/19), gentamicin 10.53% (221), doxycycline 15.79% (3/19), levofloxacin 0% (0/19), lincomycin 26.32% (5/19) resistant, and 73.68% (14/19) intermediate (Table 5).

### 3.5. Molecular Detection of Antimicrobial Resistant Associated Genes

**Figure 2:**
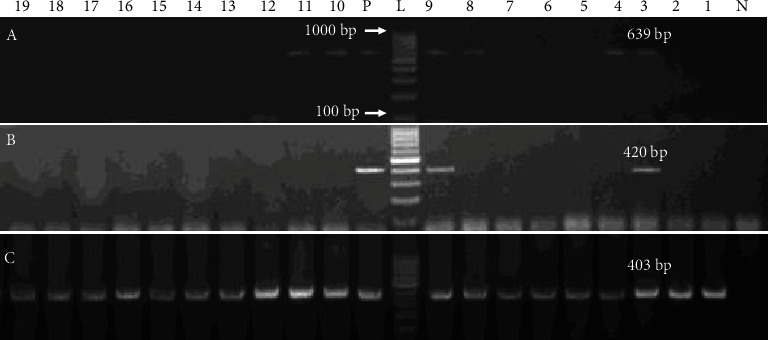
Molecular identification of macrolide resistance genes. PCR amplification of (a–c) macrolide resistance genes ermB, ereA, and mphA on an ethidium bromide-stained 1.5% TAE agarose gel. L 100 bp DNA ladder, lane P for positive control, and lane N for negative.

All the phenotypically resistant *Salmonella* isolates against ampicillin, harbored *bla*_SHV_, but 18/19 of them carried *bla*_TEM_ (Table 4 and Figure 1). Erythromycin and lincomycin-resistant strains harbored *erm*B 6/19 (31.58%), *ere*A 2/19 (10.53%), and *mph*A 19/19 (100%), respectively, as shown in Table 5 and Figure 2.

## 4. Discussion

*Salmonella species* are members of the Enterobacteriaceae family. They are nonspore-forming, facultatively anaerobic, and Gram-negative rods [8]. They pose a significant challenge in our lives nowadays. *Salmonella* spp. can be found in all poultry products that are consumed by humans, including meat and eggs. However, it can contaminate other food products and infect humans, so it is considered a health-threatening organism [39]. It is responsible for a variety of poultry diseases, including fowl typhoid, pullorum, and paratyphoid diseases. Our results showed that the clinical signs of paratyphoid *Salmonellae* including, *S*. enteritidis, *S*. *typhimurium*, *S. shangani*, *S. gueuletapee*, and S. *II salamae,* were more severe in young birds than in older ones. This may be due to a deficiency of beneficial microflora in the intestine of young chicks obtained from hatcheries, which makes them susceptible to infection with *Salmonella*. These results were consistent with [14]. In this study, 832 samples were collected from 52 poultry flocks, and we found that 19 (2.28%) of them were positive for *Salmonella* spp., and the young age was more affected by the disease than the old age, as the clinical signs and mortalities were higher at the young age than others. The isolation of *Salmonella* from the liver (3.36%) and gallbladder (2.88%) was significantly (*P* < 0.05) higher compared to that of the spleen and intestinal content (1.44%). These findings could be explained by the high invasive ability of these motile *Salmonellae*. The results found in this study were close to those of El-Sharkawy et al. [16]. Our results indicated that the isolates were serotyped as 9 (47.37%) *S. enteritidis* O 1,9,12, ad monophasic H: g, m: -, 6 (31.58.%) *S. shangani* 2, (10.53%) *S. gueuletapee* 1, (5.26%) *S. II (salamae)* and 1 (5.26%) untypable. Our results were in the same line as described by the authors of [40]. In this study, *Salmonella* spp. can be considered a major pathogen and an important hazard for the poultry industry, particularly young broilers due to the high mortalities, which showed levels of (9.64% ± 1.72) in one-week-old chicken farms compared to the none infected flocks (2.5% ± 0.99). This may be due to diarrhea dehydration, and severe lesions in the liver and other vital organs caused by infection with these motile and invasive *Salmonellae*. Our findings also showed that there was no significant difference in mortality rates between infected and uninfected 6-week-old flocks (*P*=0.15). Our results were compatible with the study conducted by El-Sharkawy et al. [16].

In the study area, ampicillin and erythromycin are the recommended first-line agents used for the treatment of poultry infections. However, these antibiotics are misused because they are not used in the right doses and durations, given the high burden of developing antimicrobial resistance strains of bacteria against these agents. This study discovered that all detected *Salmonella* strains were erythromycin- and ampicillin-resistant. Indeed, these antibiotics were the most commonly prescribed without AST. They were also the most easily available on the market without a prescription because they were also very cheap. A similar study revealed that *Salmonella* spp. was more sensitive to levofloxacin, norfloxacin, ciprofloxacin, chloramphenicol, gentamycin, streptomycin, doxycycline, and colistin, while it was more resistant to ampicillin, erythromycin, and lincomycin [41, 42].

Genes responsible for extended-spectrum*β*-lactamases (ESBL) production arise by a point mutation at the active site of the earlier *β*-lactamases and are usually plasmid-mediated. In addition, ESBL-positiveGram-negative bacteria often carry genes that confer high resistance levels to many other antibiotics [43]. This can limit the chemotherapeutic options for ESBL-producing pathogens and facilitate the interspecies and intraspecies dissemination of ESBLs. Therefore, phenotypic detection of ESBLs among Enterobacteriaceae species is important for epidemiological purposes and for limiting the spread of resistance mechanisms.

In this study, ampicillin resistance of *Salmonella* spp. was dependent on the presence of *bla*_TEM_ 18/19 (94.7%) and *bla*_SHV_ 19/19 (100%) of isolated *Salmonella.* Our finding agreed with the results described in a previously reported study by the authors of [44]. Furthermore, we found that the erythromycin resistance of *Salmonella* isolates was attributed to *erm*B 6/19 (31.58%), *ere*A 2/19 (10.53%), and *mph*A 19/19 (100%), which harbored by resistant *Salmonella* isolates. Similar results were observed by [44]. The presence of at least one of these resistance mechanisms in all resistant strains may have been responsible for an increasing number of mortalities in one-week-old broiler chicken farms.

## 5. Conclusion

This study has been focused on giving a clear pattern of the current situation of *Salmonella* spp. infection in broiler chickens, especially in Egypt. *Salmonella* spp., including prevalence, serotyping, and an antimicrobial resistance profile. As a result, it is prudent for farmers to develop and share knowledge about salmonellosis diagnosis, treatment, and prevention protocols in order to reduce economic losses and human health risks. Limiting disease burdens would not only improve the well-being of managed broilers but also provide new avenues for achieving the WHO's global development goal of eliminating poverty and famine.

## Figures and Tables

**Table 1:** Oligonucleotide primer sequences and their corresponding genes are used for the detection of antimicrobial-resistant genes in *Salmonella* isolates.

Gene	Primer sequence (5′-3′)	Length of amplified product	Annealing temperature (°C)	Reference
*mphA*	GTGAGGAGGAGCTTCGCGAG	403 bp	58	[34]
	TGCCGCAGGACTCGGAGGTC			
*ereA*	GCCGGTGCTCATGAACTTGAG	420 bp	60	
	CGACTCTATTCGATCAGAGGC			
*ermB*	GAAAAAGTACTCAACCAAATA	639 bp	45	
	AATTTAAGTACCGTTACT			

*bla *_TEM_	ATCAGCAATAAACCAGC	516 bp	54	[35]
	CCCCGAAGAACGTTTTC			
*bla *_SHV_	AGGATTGACTGCCTTTTTG	392 bp	54	
	ATTTGCTGATTTCGCTCG			

*ompC F*	ATCGCTGACTTATGCAATCG	204 bp	57	[33]
*ompC R*	CGGGTTGCGTTATAGGTCTG	204 bp		

**Table 2:** Breakpoint values of each antimicrobial agent according to [36].

Antimicrobial agent(s) tested	Disc concentration	*Salmonella* isolates		
		Resistant ≤ mm	Intermediate (mm)	Sensitive ≥ mm
Ampicillin (AM)	10 μg	13	14 : 16	17
Ciprofloxacin (CIP)	5 μg	15	16 : 20	21
Chloramphenicol (C)	30 μg	12	13 : 17	18
Streptomycin (STR)	10 μg	11	12 : 14	15
Gentamycin (CN)	10 μg	12	13 : 14	15
Erythromycin (E)	15 μg	13	14 : 22	23
Doxycycline (Do)	30 μg	10	11 : 13	14
Levofloxacin (LEV)	5 μg	13	14 : 16	17
Lincomycin (L)	2 μg	9	10 : 14	15
Norfloxacin (NOR)	10 μg	12	13 : 16	17
Colistin (CT)	10 μg	10	11 : 13	14

**Table 3:** Putative *Salmonella* spp. that was isolated from the organs of individual chickens.

Organ	Liver	Gall bladder	Spleen	Intestine	Total
No. of collected samples	208	208	208	208	832
*S. enteritides*	3 (1.44%)	3 (1.44%)	2 (0.96%)	1 (0.48%)	9 (1.08%)
*S. shangani*	2 (0.96%)	2 (0.96%)	1 (0.48%)	1 (0.48%)	6 (0.72%)
*S. gueuletapee*	1 (0.48%)	1 (0.48%)	0	0	2 (0.24%)
*S. II (salamae)*	1 (0.48%)	0	0	0	1 (0.12%)
Unconformity	0	0	0	1 (0.48%)	1 (0.12%)
TOTAL	7 (3.36%)	6 (2.88%)	3 (1.44%)	3 (1.44%)	19 (2.28%)

**Table 4:** : Results of the serological identification of *Salmonella* isolates.

Serotype	Number	O Antigen		H Antigen
			Phase 1	Phase 2
*Salmonella enteritidis*	9	1, 9, 12 g, m	g, m	—
*Salmonella shangani*	6	3, 10, 15	d	1, 5
*Salmonella gueuletapee*	2	9, 12	g, m, s	—
*Salmonella II (salamae)*	1	6, 8	g, *s*, t	e, n, x
Untypable *Salmonella* spp.	1	—	—	—

**Table 5:** Results of the antibiotic sensitivity test for *Salmonella spp.* and the PCR that was performed to detect the resistance genes.

Antibiotic disk sample no.	Serotype	CIP	NOR	C	S	L	E	AM	CN	LEV	DO	CT	*ereA*	*ermB*	*mphA*	*bla*_TEM_	*bla*_SHV_
1	*S.*	S	S	S	S	R	R	R	R	S	I	*I*	−	−	+	+	+
2	*S.*	S	S	S	I	I	R	R	S	S	R	R	−	−	+	+	+
3	*S.*	S	S	S	R	R	R	R	S	S	R	R	+	+	+	+	+
4	*S.*	S	S	S	S	R	R	R	S	S	I	S	−	+	+	+	+
5	*S.*	S	S	S	I	R	R	R	S	S	R	I	−	−	+	+	+
7	*S.*	S	S	I	I	R	R	R	S	S	I	R	−	−	+	+	+
8	*S.*	S	S	S	S	I	R	R	S	S	I	S	−	−	+	+	+
9	*S.*	S	S	I	S	I	R	R	S	S	S	I	−	+	+	+	+
10	*S.*	S	S	S	S	I	R	R	*I*	S	I	R	+	+	+	+	+
12	*S.*	S	S	S	I	I	R	R	*I*	S	s	S	−	+	+	+	+
13	*S.*	S	S	S	S	I	R	R	S	S	s	S	−	+	+	+	+
14	*S.*	S	S	S	S	I	R	R	S	S	I	I	−	−	+	+	+
15	*S.*	S	S	s	S	I	R	R	R	S	s	S	−	−	+	+	+
16	*S.*	S	S	s	R	I	R	R	S	S	s	R	−	−	+	+	+
17	*S.*	S	S	S	S	I	R	R	S	S	S	S	−	−	+	+	+
18	*S.*	S	S	I	S	I	R	R	S	S	I	S	−	−	+	−	+
19	*S.*	S	S	S	S	I	R	R	S	S	S	I	−	−	+	+	+

## Data Availability

The data used to support the findings of this study are available from the corresponding author upon reasonable request.
